# Neural correlates of social influence on risk perception during development

**DOI:** 10.1080/17470919.2020.1726450

**Published:** 2020-02-24

**Authors:** L. J. Knoll, A. Gaule, A. Lazari, E. A. K. Jacobs, S. J. Blakemore

**Affiliations:** a University College London, London, WC, UK; b Wellcome Centre for Integrative Neuroimaging, FMRIB, Nuffield Department of Clinical Neurosciences, University of Oxford, UK; c Department of Psychology, University of Cambridge, UK

**Keywords:** Adolescence, social influence, development, fMRI, neuroimaging, social conflict

## Abstract

Studies have shown that adolescents are more likely than adults to take risks in the presence of peers than when alone, and that young adolescents’ risk perception is more influenced by other teenagers than by adults. The current fMRI study investigated the effect of social influence on risk perception in female adolescents (aged 12–14) and adults (aged 23–29). Participants rated the riskiness of everyday situations and were then informed about the (alleged) risk ratings of a social influence group (teenagers or adults), before rating each situation again. The results showed that adolescents adjusted their ratings to conform with others more than adults did, and both age groups were influenced more by adults than by teenagers. When there was a conflict between the participants’ own risk ratings and the ratings of the social influence group, activation was increased in the posterior medial frontal cortex, dorsal cingulate cortex and inferior frontal gyrus in both age groups. In addition, there was greater activation during no-conflict situations in the right middle frontal gyrus and bilateral parietal cortex in adults compared with adolescents. These results suggest that there are behavioral and neural differences between adolescents and adults in conflict and no-conflict social situations.

## Introduction

Being part of a social group and engaging in social interactions allows us to learn about the world through other people’s eyes, without necessarily experiencing a situation first-hand (Frith & Frith, 2007). Human decision-making is influenced by other people in that other people’s beliefs and actions can have a large impact on our own behavior (Berns, Capra, Moore, & Noussair, 2010). For example, we tend to conform to group behavior even when it conflicts with our own beliefs or perceptions (Asch, 1951; Cialdini & Goldstein, 2004). It has been proposed that this social influence effect is due to the pursuit of acceptance by others as well as the belief that others’ behavior is more accurate than our own (Deutsch & Gerard, 1955).

Age is an important factor in determining the degree of susceptibility to social influence. Adolescence, the period of life between puberty onset and adult independence, is a developmental stage in which people can be particularly susceptible to social influence. Studies have demonstrated a higher susceptibility to social influence in young adolescents than in adults (Costanzo & Shaw, 1966; Hoving, Hamm, & Galvin, 1969; Knoll, Leung, Foulkes, & Blakemore, 2017; Knoll, Magis-Weinberg, Speekenbrink, & Blakemore, 2015). In our previous behavioral studies, we included large groups of participants aged 8 to 59 years, and asked them to rate the riskiness of everyday situations. Participants were then informed about the risk ratings of a (fictitious) social influence group comprising either *adults* or *teenagers* and were then asked to rate the same situation again. We found that the degree to which participants changed their ratings to conform with those of other people decreased from late childhood to adulthood. Most age groups (children, young adults and adults) were more influenced by the social influence group *adults* than by *teenagers*. Mid-adolescents (aged 15–18) were similarly influenced by *adults* and *teenagers*. In contrast, young adolescents (aged 12–14) adjusted their risk ratings more to conform with *teenagers* than with *adults*, suggesting that young adolescents are more susceptible to be influenced by people their own age than by adults. A subsequent study (Knoll et al., 2017) using the same paradigm found that children (aged 8–11) and young adolescents (aged 12–14) were more influenced by *teenage* feedback, but only when *teenagers* apparently rated the situation as riskier than the participants. The findings suggest that socially shared expectations of stereotypes about specific groups, such as risk-prone teenagers, affect the degree of conforming behavior. These findings are in line with previous studies which, for example, reported that during adolescence risky decisions in a driving game increased in the presence of peers compared with when alone, while the presence of peers did not affect adults’ driving behavior (Gardner & Steinberg, 2005). Risks such as smoking, experimenting with drugs and alcohol, and dangerous driving, are all more likely to occur in social contexts than when alone in adolescence (Blakemore, 2018).

Several neuroimaging studies in adults have investigated social influence in situations in which other people’s behavior conflicts with the behavior of the participant. Convergent evidence from recent studies has highlighted the role of the posterior medial frontal cortex (pMFC) in performance monitoring and making appropriate behavioral adjustments (Ridderinkhof, Ullsperger, Crone, & Nieuwenhuis, 2004). The pMFC appears to play an important role in processing situations of social conflict. For example, activation in the pMFC is associated with social conformity – adjusting one’s own opinion toward that of a group – following conflicting information when participants rate the attractiveness of faces (Klucharev, Hytönen, Rijpkema, Smidts, & Fernández, 2009). More causal evidence of a role for the pMFC in social conflict comes from a study showing that social conformity behavior is reduced following down-regulation of the pMFC via TMS (Klucharev, Munneke, Smidts, & Fernández, 2011). It has been suggested that the pMFC facilitates regulatory processes to adjust behavior to changes in the environment, especially negative changes such as response conflict (Izuma & Adolphs, 2013; for a review see Ridderinkhof et al., 2004). The dorsomedial prefrontal cortex (dmPFC), a region within the pMFC, was found to be sensitive to the *source* of the conflicting information (Izuma & Adolphs, 2013). When participants were shown clothing preferences purporting to be either from fellow students or from sex offenders, activation in the dmPFC increased when the *liked* group showed a different preference from the participant or when the *disliked* group showed a similar preference as the participant. This suggests that the dmPFC is sensitive to social conflict as well as how participants feel toward the social influence group.

In contrast, agreement with group opinions tends to be associated with activation in reward-related brain regions in adults (Berns et al., 2010; Campbell-Meiklejohn, Bach, Roepstorff, Dolan, & Frith, 2010; Izuma & Adolphs, 2013). In one study participants were asked to choose between two songs and were then informed which of the two songs someone else preferred (Campbell-Meiklejohn et al., 2010). Agreement between the other person and the participant was associated with increased activation in reward related regions of the brain such as the ventral striatum. The nucleus accumbens (NAcc), which is part of the ventral striatum, has been found to be activated when participants adapted their behavior to conform with others (Klucharev et al., 2009). In comparison with adults, the NAcc seems to be hypersensitive to reward in adolescence and its activity has been found to peak during this time (Braams, van Duijvenvoorde, Peper, & Crone, 2015; Galvan et al., 2006). In a study in which participants had to make a simple judgment (indicating the side of the screen) of sequential cues representing small medium and large rewards in an fMRI scanner, Galvan and colleagues (2006) found the NAcc to be more sensitive to reward processing and evaluation in adolescents than in children or adults.

Whereas studies have investigated reward processing and peer influence on decision-making in adolescence, less is known about the mechanisms supporting social influence itself (Welborn et al., 2016). Given previous behavioral and neural findings demonstrating that adolescents are particularly susceptible to peer influence (Chein, Albert, O’Brien, Uckert, & Steinberg, 2011; Knoll et al., 2017, 2015), we were interested in investigating how the source of socially conflicting information is associated with brain activity during social influence between adolescence and adulthood.

The aim of the current study was to investigate the neural mechanisms of social influence on risk perception in adolescents and adults, using an adapted version of our social influence paradigm in which other people’s risk perception was either in conflict or agreement with participants’ own risk perception (Knoll et al., 2017, 2015). As in our behavioral paradigm, participants were asked to rate the riskiness of everyday situations and were then presented with (fictitious) risk ratings of the same situations from other people, either *teenagers* or *adults*. These fictitious ratings either conflicted or did not conflict with the participant’s original rating. Participants were subsequently asked to rate the same situation again. The paradigm allowed us to assess the behavioral and neural effects of conflicting and non-conflicting feedback on a trial-by-trial basis. We investigated four hypotheses:

H1. Adolescents would show a higher social influence effect than adults, and this might be especially pronounced when the social influence group was *teenagers*, as we found previously (Knoll et al., 2017, 2015). We also predicted that adolescents would change their responses under conflict more than adults (Knoll et al., 2017).

H2. When the provided rating of the social influence group is in conflict with the initial rating of the participants, we expected to find increased activation in the pMFC, similar to that observed in studies investigating conformity following social conflict (Klucharev et al., 2011; Ridderinkhof et al., 2004).

H3. When the provided rating of the social influence group is not in conflict with the initial rating of participants, we predicted activation in reward-related regions (in particular the NAcc), and that this effect would be greater in adolescents than in adults.

H4. As the dmPFC is sensitive to behavioral adjustment away from a disliked group toward liked groups (Izuma & Adolphs, 2013), we predicted that this region might be sensitive to the social influence group. In this previous study, the liked group was defined as fellow peers and the disliked group was a group of sex offenders. This is more extreme than in the current study, but our hypothesis is that adolescents identify themselves more with the teenage influence group than the adult influence group, and adults more with the adult influence group than with the teenage influence group.

## Material and methods

### Participants

Twenty-two female adolescents (aged 12–14) and 20 female adults (aged 23–29) took part in this fMRI experiment. Data from four adolescents and one adult were excluded due to movement artifacts and from one adult who expressed doubts about the paradigm manipulation (see Supplemental Information for protocol, A). The final sample for the fMRI experiment consisted of 18 adolescent participants (mean age 13.33 years, range 12 to 14 years) and 18 adult participants (mean age 24.06 years, range 23–29 years). All participants were female in order to avoid sex difference-related confounds within the sample. All participants were English speakers, had no neurological, medical, or psychological disorders, and no contraindications to obtaining an MRI scan. Study procedures were approved by the local Research Ethics Committee. Adult participants, and parents or legal guardians of adolescent participants, gave informed consent for the study. Participants were reimbursed at a rate of £10 per hour for taking part.

Verbal IQ was estimated using the verbal subtest of Wechsler’s Abbreviated Scale of Intelligence (Wechsler et al., 1999) and standardized for each age group. There was no significant difference between adolescent vIQ (mean: 59.61; sd: 7.4) and adult vIQ (mean: 58.44; sd: 9.1; t(34) = −.440; *p* = 0.663).

### Study design

The fMRI study employed an event-related design with two within-subject factors: Social influence group (*teenagers* vs *adults*) and conflict (*conflict* vs *no-conflict*); and one between-subjects factor: age group (*adolescents* vs *adults*).

**Figure 1.**
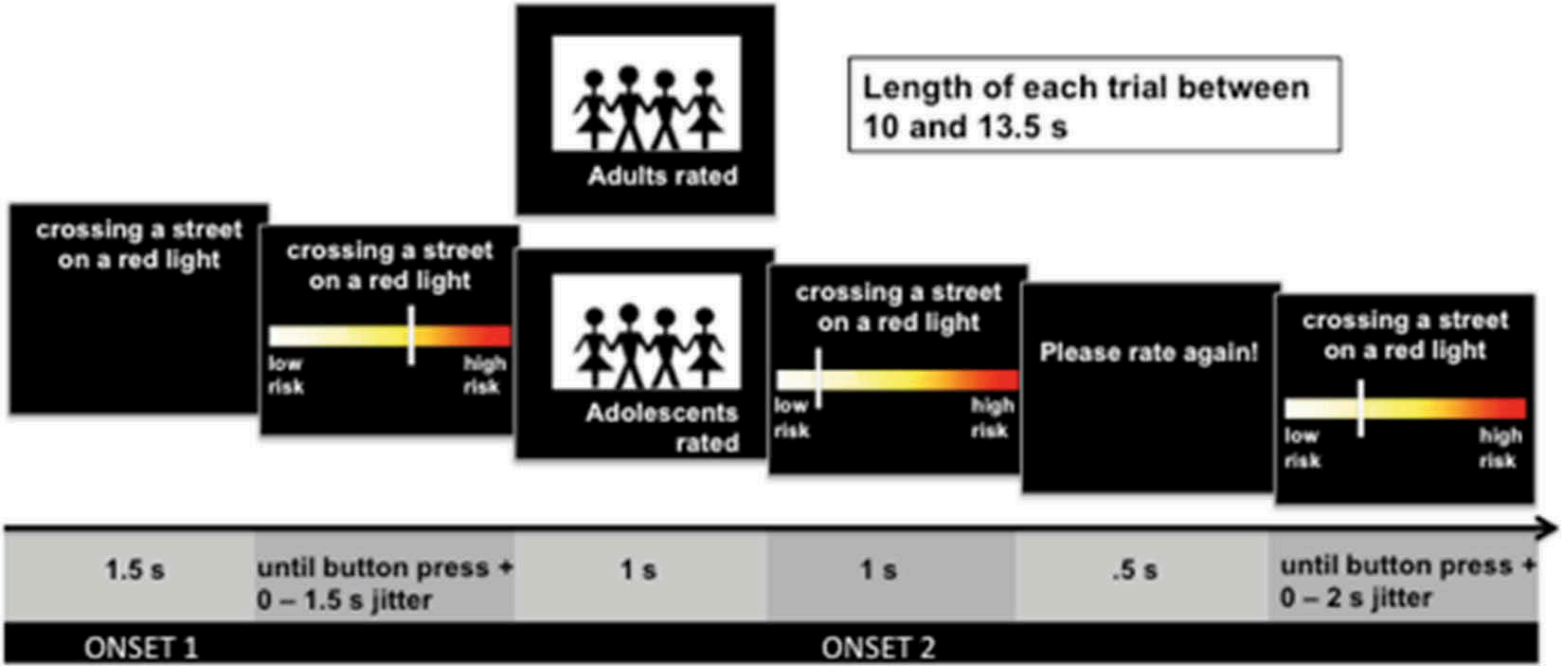
Experimental design. Presentation of an everyday situation (1.5 s). Risk rating from low risk to high risk (4 s time limit). Presentation of risk rating of a (fictional) social influence group (1 s): Teenagers (in the experimental design labeled as adolescents) or adults. Second risk rating (4 s time limit). Each trial started after a random jitter between 0 and 2 s.

We used an adapted version of our social influence on risk-perception task (Knoll et al., 2017, 2015). Participants were presented with four blocks of 40 trials (total 160 trials), each of which depicted a risky scenario. The order of scenario presentation was randomized for each participant. Stimuli consisted of single statements, e.g. “Crossing a street on a red light,” (see Supplemental Information, material D) and were displayed at the top of a screen for 1.5 s. Participants were asked to imagine someone engaging in the activity presented and then rated the activity’s riskiness by using their right index finger to move a slider to the left side (low risk) or right middle finger to move the slider to the right side (high risk) of a visual analogue scale (see Figure 1). The full range of the analogue scale was 0–10. The slider initially appeared at a random position on the scale on each trial to avoid any systematic anchoring bias. Participants had 4.5 s to confirm the rating by pressing a button with their left index finger. After providing the first rating, participants were shown a risk rating of the same situation that they were told was the average of a group of either adults or teenagers (the social influence group), who previously took part in our behavioral study in the Science Museum. A cue (1 s) indicated whether the subsequent rating was from either teenagers or adults, which was followed by the rating (1 s).

These provided ratings were ostensibly from other participants; in fact, they were programmed such that for each participant, four blocks of 40 trials (total 160 trials) of the risk perception task were generated. In each block, 20 scenarios per social influence group were presented in an event-related, pseudo-randomized design (no more than two consecutive trials belonging to the same social influence group were allowed). In 40 trials ratings for the social influence group were lower than the participant’s initial rating (deviated by −2 or more points), and in 40 trials they were higher (deviated by +2 or more points). These trials represented the conflict condition. In 80 trials they were similar to the participant’s first rating (deviated by 0 to 1 points). These trials represented the no-conflict condition. This minor deception was approved by the ethics committee.

After the provided rating was shown, the sentence “Please rate again!” was presented for 0.5 s and participants were asked to rate the same situation again (see Figure 1). There was a 4.5 s time restriction for the second rating, as for the first rating. The subsequent trial started after a 0–2 s jitter after participants confirmed their second rating. If no rating was made within the time limit, a red cross was presented on the screen for 2 s and the next trial started. Trials in which participants missed a rating were excluded from the analysis. For Rating 1, adolescents missed 29 ratings and adults missed 18 ratings. For Rating 2, adolescents missed 6 ratings and adults missed 8 ratings.

### Procedure

Before the fMRI experiment started, the participant was familiarized with the experimental setting and underwent a training session in a testing room. The training paradigm employed the same experimental design as the main experiment in terms of timings and ratings. However, instead of risk ratings, participants were asked to rate how much they liked a food option and were then shown the likability rating of the same food option by either Ernie or Bert from Sesame Street. After that they were asked to rate the food option a second time.

In the scanner, the participant performed four blocks of 40 trials of the risk perception task. Each block lasted approximately 8 minutes, and participants were given a short break between each block. After the second experimental block, the structural data were acquired.

After the fMRI experiment, participants carried out the verbal IQ test and completed the resistance to peer influence questionnaire (RPI, Steinberg & Monahan, 2007) and the future orientation scale (FOS, Steinberg et al., 2009), as previous studies have demonstrated age differences in these relevant measures. There were however no significant differences between the RPI score of adolescents (mean: 2.94; sd: 0.21) and adults (mean: 2.91; sd: 0.63) (t(24.58) = .263; *p* = 0.79), or between the FOS score of adolescents (mean: 3.03; sd: 0.40) and adults (mean: 3.11; sd: 0.45) (t(34) = −.563; *p* = 0.58).

The entire testing procedure, including training, IQ testing, questionnaires, and fMRI data acquisition, took around 2 hours.

Between two to eight weeks after the study, participants were contacted by phone and debriefed (see Supplementary Information for protocol, A). They were asked to tell us about the experiment and how they had perceived the ratings of the social influence group in order to check they believed the manipulation. Participants were then informed that the ratings of the social influence group were not real but actually were computer-generated. One adult participant questioned the paradigm and whether the presented risk ratings were real; her data were excluded from the analysis.

### Fmri data acquisition

Brain imaging data were acquired on a Siemens Avanto 1.5 T MRI scanner (Erlangen, Germany). Functional data were acquired in four sessions each lasting approximately 8 minutes with a multi-slice T2*- weighted echo-planar sequence with blood oxygenation level-dependent (BOLD) contrast (repetition time (TR) 3 s, echo time (TE) 50 ms). In each session, between 133 and 175 volumes were sampled and each volume comprised 35 axial slices (in-plane resolution: 3x3x3 mm) covering most of the cerebrum. The task was presented and responses were acquired with Cogent 2000 (www.vislab.ucl.ac.uk/Cogent/index.html) using Matlab R2010b (Mathwork Inc. Sherborn, MA). Stimuli were front-projected onto a screen, which participants viewed via a mirror mounted on the head coil. Structural data were acquired with a T1-weighted fast-field echo structural image sequence lasting 5 min 30 s. Functional imaging data were preprocessed and analyzed using SPM12 (Statistical Parametric Mapping, Wellcome Trust Center for Neuroimaging, http://www.fil.ion.ucl.ac.uk/spm/). To allow for T1 equilibration effects, the first four volumes of each session were discarded. Images were realigned to the first analyzed volume with a second-degree B-spline interpolation to correct for movement during the session. The data were then slice-time corrected to the middle-slice. The bias-field corrected structural image was co-registered to the mean, realigned to the functional image and segmented on the basis of Montreal Neurological Institute (MNI)-registered International Consortium for Brain Mapping (ICBM)-tissue probability maps. Resulting spatial normalization parameters were applied to the realigned images to obtain normalized functional images with a voxel size of 3x3x3 mm^3^, which were smoothed with an 8-mm full width at half maximum (FWHM) Gaussian kernel.

Realignment estimates were used to calculate frame-wise displacement (FD) for each volume, which is a composite, scalar measure of head motion across the six realignment estimates (Siegel et al., 2014). Volumes with an FD 0.9 mm were censored and excluded from general linear model (GLM) estimation by including a regressor of no interest for each censored volume. Scanning sessions with more than 10% of volumes censored or a root mean square (RMS) movement over the whole session greater than 1.5 mm were excluded from the analysis. Adolescent and adult participants significantly differed in RMS (adolescents = 0.35 mm, sd = 0.12; adults = 0.29 mm, sd = 0.11; t(1) = 9.61, p < 0.01) and mean FD (adolescents = 0.23 mm; sd = 0.16; adults = 0.11 mm, sd = 0.05; t(1) = 7.67, p < 0.01) and number of censored scans (adolescents = 393; adults = 33; t(1) = 19.46, p < 0.001). Based on the defined criteria, one session from one adult participant and six sessions from a total of five adolescent participants were excluded from analysis.

### Behavioral data analysis

All statistical models were estimated in R (R Core Team, 2004), using the lme4 (Bates, Maechler, & Bolker, 2013) and lmerTest (Kuznetsova, Brockhoff, & Christensen, 2013) packages. We applied a linear mixed-effects model to investigate the degree to which participants changed their risk ratings in the direction of other people’s ratings when there was no conflict between the first risk rating of the participant and the provided rating of the social influence group (*no-conflict*) and when the provided rating was either more or less risky than the first rating of the participant (*conflict*), and the extent to which this change depended on whether the social influence group consisted of *adults* or *teenagers*. This model incorporated (a) fixed effects that reflected average effects within and differences between the experimental conditions and (b) random effects that took into account individual variability and scenario-specific variability for the first rating and slider position post feedback of the social influence group.

The linear mixed-effects model assessed the dependency of participant’s change in rating (absolute difference between rating 1 and rating 2) on age (binary independent variable), conflict (binary independent variable) and the interaction of age and conflict, age and social influence group and age, conflict and social influence group.

### Fmri data analysis

Using SPM12, the statistical evaluation was based on a least-squares estimation using the general linear model for serially autocorrelated observations (Friston, Jezzard, & Turner, 1994). The design matrix was generated with a synthetic hemodynamic response function (Josephs, Turner, & Friston, 1997). Data were acquired in four sessions, which were included in the model as separate time series and each series was modeled by a set of regressors in the first level analysis. Each censored volume was included in the model as separate regressor. Data were high-pass filtered (128 s). The first level analysis included eight event-related regressors: four regressors at OT1 (OT1 = onset time of scenario presentation without the rating of social influence group) reflecting four task conditions (*adults/no-conflict, adults/conflict, teenagers/no-conflict, teenagers/conflict*) as well as four regressors at OT2 (OT2 = onset time of scenario presentation with rating of the social influence group) reflecting the above mentioned four task conditions. These parameter estimates were used to create four contrasts comparing each of the task conditions (two social influence group x two conflict) at OT2 to the respective task condition at OT1. These four contrasts were then entered into a random-effects analysis using a subject x age group x social influence group x conflict flexible factorial design, modeling factors as main effects (the subject factor was included to account for the repeated-measure nature of the data) and a social influence group x conflict interaction. First, we investigated the main effects of conflict (*conflict > no-conflict, no-conflict > conflict*) and social influence group (*teenagers > adults, adults > teenagers*) and the interaction between social influence group and conflict. Second, to investigate age group differences, we created first level contrast images of conflict (*conflict > no-conflict, no-conflict > conflict*) and social influence group (*teenagers > adults, adults > teenagers*) and the interaction between social influence group and conflict. We then ran a t-test at the second level to compare age groups (adults, adolescents). To protect against false-positives, reported results were thresholded at voxel-level p < 0.001 uncorrected and cluster-level p < 0.05 family wise error [FWE] corrected. Finally, we used Marsbar (Brett, Anton, Valabregue, & Poline, 2002) to create a 5 mm sphere around the NAcc, using the coordinates used by Klucharev et al. (2009) (x = ±11, y = 11, z = −2) and extracted the estimates to analyze the interaction between social influence group and conflict.

## Results

### Behavioral data

**Figure 2.**
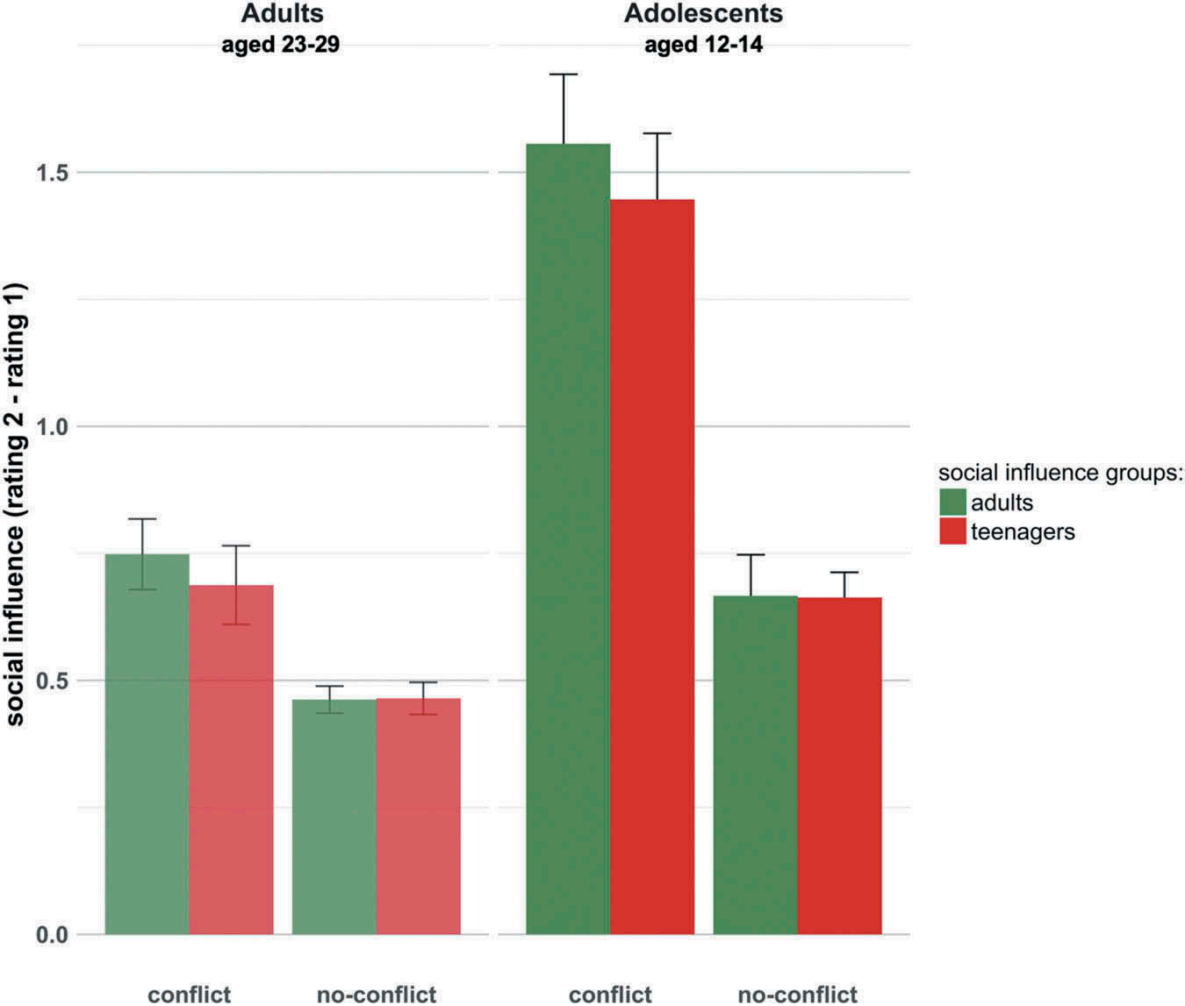
The graph shows the average differences in rating (rating 2 minus rating 1) with standard error bars. Results are shown separately for the adult social influence (green bars) and teenage social influence (red bars) conditions, and for the conflict and no-conflict conditions. We found significant interaction effects between age group and conflict (Χ^2^(1) = 186.08, p < 0.001), indicating that adolescents changed their responses more under conflict than did adults. We also found a significant interaction between conflict and social influence, indicating that participants responded more to adult feedback in conflict trials (Χ^2^(2) = 6.66, p < 0.05). There was no significant interaction between social influence group and age group, and no three-way interaction between age group, social influence group and conflict.

We used a linear mixed-effects model to investigate whether the two age groups (adolescents and adults) changed their ratings under *conflict* and *no-conflict* conditions, and whether this change depended on whether the social influence group consisted of *adults* or *teenagers*. There was a significant main effect of conflict (Χ^2^(1) = 648.79, p < 0.001), with participants changing their responses more toward ratings of the social influence group under conflict. There was also a main effect of age group (Χ^2^(1) = 103.61, p < 0.001), driven by teenagers changing their responses more generally more than adults. There was a significant interaction between age group (of participant) and conflict (Χ^2^(2) = 186.09, p < 0.001), driven by adolescents changing their responses more under conflict than adults. Finally, there was a significant interaction between conflict and social influence (Χ^2^(2) = 6.66, p < 0.05) whereby participants changed their ratings more to *adult* feedback under *conflict*. There was no three-way interaction between age group, social influence group and conflict. Results are summarized in Figure 2 (see Supplementary Material for table).

#### Fmri results

##### Main effect of conflict

**Figure 3.**
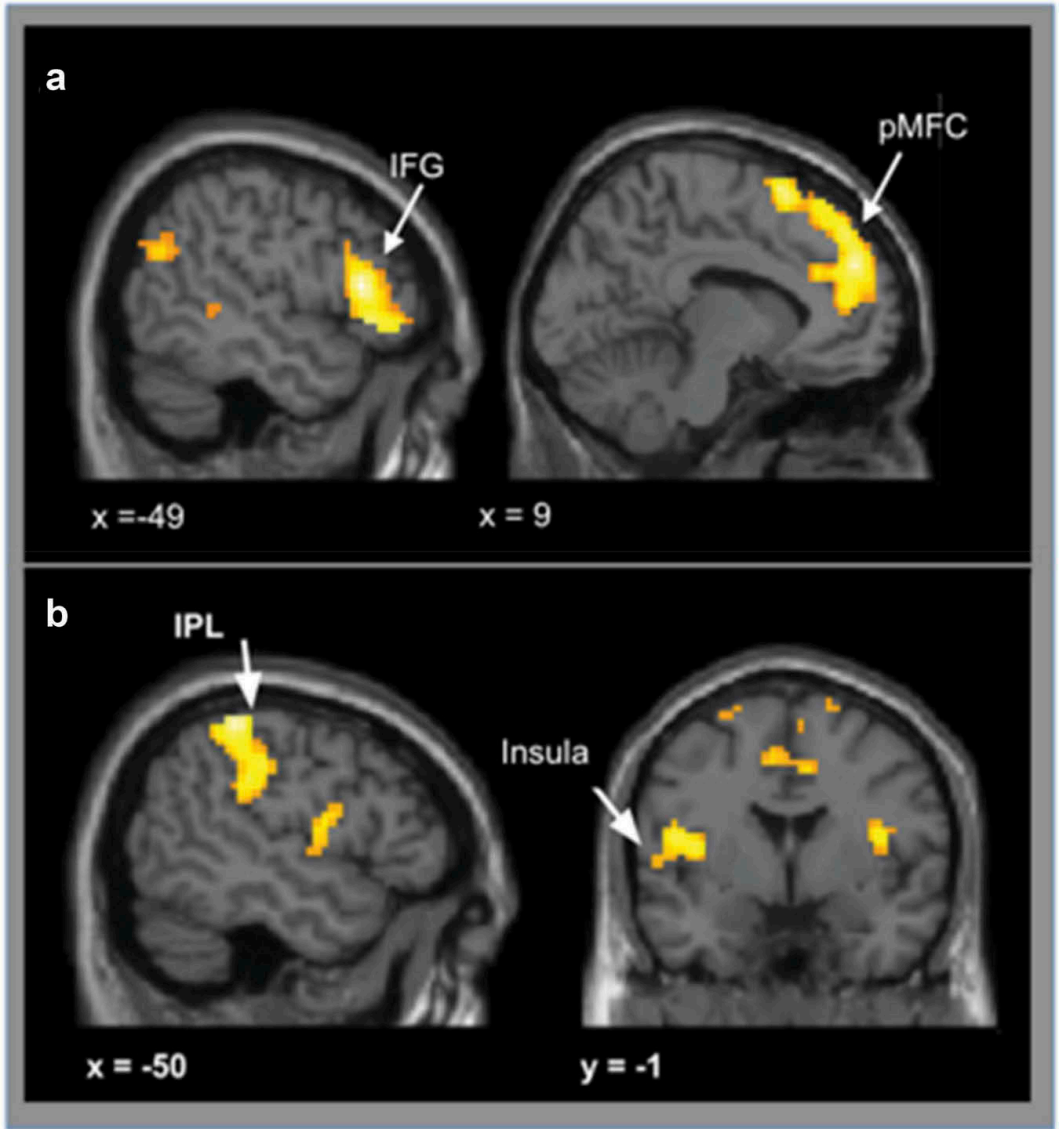
A shows activation for conflict vs no-conflict trials. When participants were presented with conflict conditions, we observed greater bilateral activation in posterior medial frontal cortex (pMFC), inferior frontal gyrus (IFG) and left dorsal ACC compared to no-conflict versus conflict conditions. B shows activation for no-conflict vs conflict trials. Compared to conflict trials, no-conflict trials lead to stronger activation in the insula, bilaterally, the left posterior cingulate cortex (PCC), left parietal lobe (IPL) and precentral regions. Results were cluster level corrected (p < 0.05, voxel-level uncorrected p < 0.001, cluster level corrected at p_FWE_ < 0.05).

*Conflict vs. no-conflict*: When participants were presented with *conflict* vs *no-conflict* conditions (collapsing over social influence group), there was increased activity in three clusters, including the bilateral pMFC, with peak activity in the supplementary motor area (SMA), and inferior frontal gyrus cluster (IFG) that included the anterior insular (AI), as well as the left dorsal ACC (see Figure 3(a), Table 1).

**Table 1.** No-conflict > conflict (voxel-level uncorrected p < 0.001, cluster level corrected at p_FWE_ < 0.05).

				Peak Voxel (in mm)		
Cluster	Brain Region	Size (N voxels)	z	**x**	**y**	**z**
Left Parietal cluster	**Parietal Inferior Lobule (IPL)**	**600**	**5.84**	**−42**	**−46**	**50**
	Postcentral gyrus (PoCG)		5.29	−51	−37	56
Right Parietal cluster	**Supramarginal gyrus (SMG)**	**784**	**5.48**	**54**	**−19**	**26**
	Postcentral gyrus (PoCG)		5.18	42	43	59
Right Insula	**Insula (I)**	**59**	**4.76**	**39**	**5**	**11**
Left Insula	**Rolandic Operculum (ROL)**	**90**	**4.61**	**−48**	**−1**	**14**
	Insula (I)		4.53	−39	−1	11
Left Frontal	**Posterior Cingulum (PCG)**	**68**	**4.55**	−**15**	**−40**	**14**
Left SMA	**Precentral (PreCG)**	**282**	**4.36**	**−36**	**−13**	**65**
	Supplementary Motor Area (SMA)		4.10	−15	−10	62

*No-conflict vs. conflict*: When participants were presented with *no-conflict* vs *conflict* trials (collapsing over social influence group), activation was observed bilaterally in the posterior insula, left posterior cingulate cortex (PCC), left parietal cortex and precentral gyrus (see Figure 3(b), Table 2).

**Table 2.** Conflict > no-conflict (voxel-level uncorrected p < 0.001, cluster level corrected at p_FWE_ < 0.05).

				Peak Voxel (in mm)		
Cluster	Brain Region	Size (N voxels)	z	**x**	**y**	**z**
Left IFG cluster	**Inferior frontal gyrus (IFGtriang)**	**427**	**6.19**	**−51**	**23**	**14**
	Inferior frontal gyrus (ORBinf)		5.60	−33	26	−4
Left pMFC cluster	**Supplementary Motor Area (SMA)**	**1314**	**5.93**	**−6**	**17**	**59**
	Medial superior frontal gyrus (SFGmed)		5.73	−9	50	29
	Anterior Cingulum		5.47	−3	47	14
Right IFG cluster	**Inferior frontal gyrus (ORBinf)**	**219**	**5.24**	**45**	**32**	**−7**
	Inferior frontal gyrus (IFGtri)		5.10	54	23	11
	Anterior Insula (AI)		4.10	27	17	−16
Left Parietal	**Angular (ANG)**	**59**	**4.17**	**−45**	**−64**	**−26**
Left Temporal	**Middle Temporal Gyrus (MTG)**	**79**	**3.68**	**−57**	**−46**	**−1**

##### Main effect of social influence group

*Teenagers* vs. *adults*: In the trials in which the social influence group was *teenagers* compared to *adults* (collapsing over conflict), there was activation in the right amygdala (see Table 3).

**Table 3.** Teenagers > adults (social influence group) (voxel-level uncorrected p < 0.001, cluster level corrected at p_FWE_< 0.05).

				Peak Voxel (in mm)		
Cluster	Brain Region	Size (N voxels)	z	**x**	**y**	**z**
**Amygdala**	**Amygdala**	**66**	**4.37**	**27**	**1**	**−19**
	Pallidum		3.56	24	−7	−7

*Adults* vs. *teenagers*: There was no significant activation for the contrast *adults* compared to *teenagers*.

##### Interaction between age group and conflict

**Figure 4.**
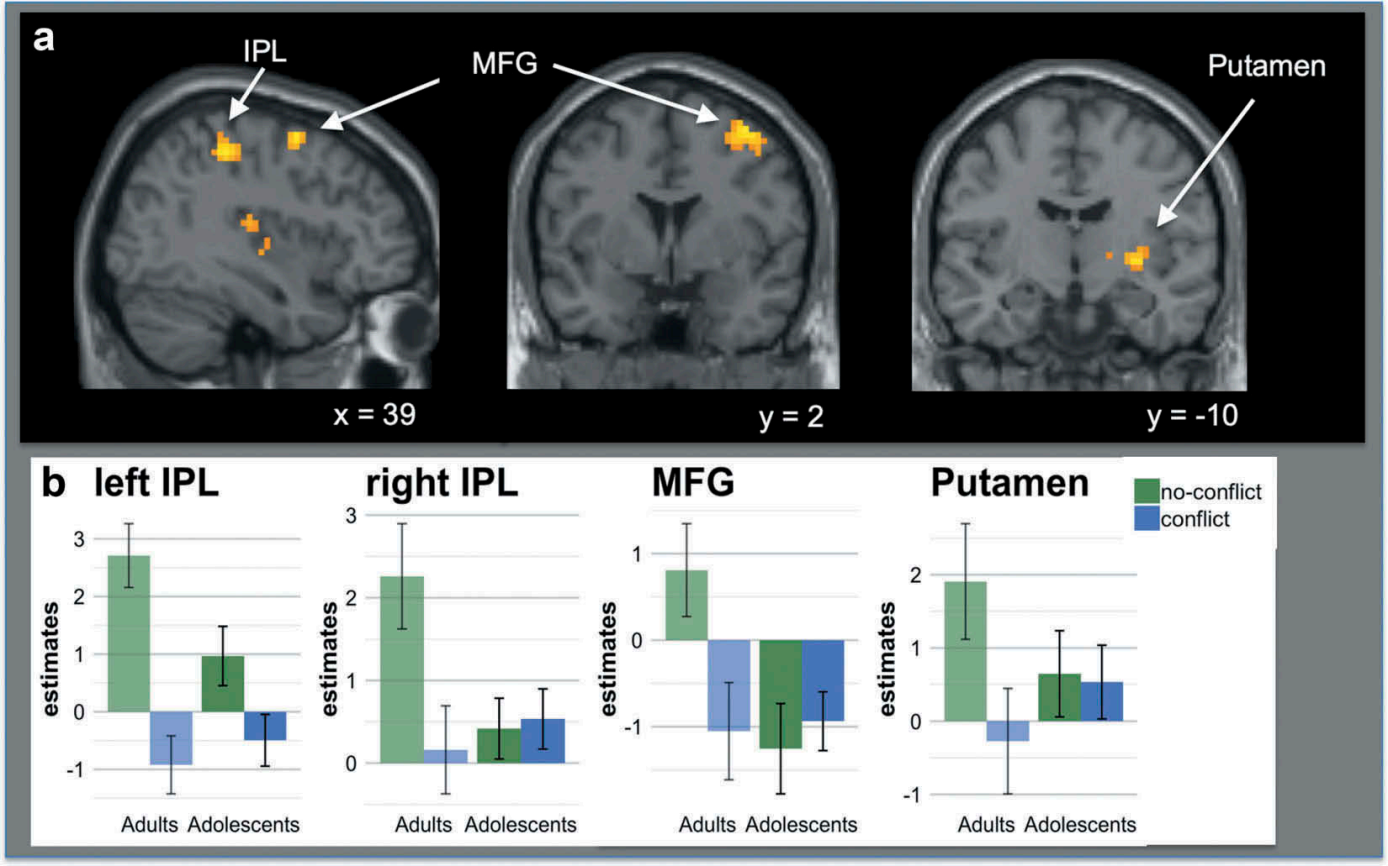
A shows significant activation for the interaction between conflict and age group. Adults showed increased activation during social agreement in the bilateral parietal cortex (IPL), right middle frontal gyrus (MFG) and putamen, compared to adolescent participants (see Table 4). Results were cluster level corrected (p < 0.05, voxel-level uncorrected p < 0.001, cluster level corrected at p_FWE_ < 0.05). B shows estimates for the four observed clusters (bilateral IPL, MFG and putamen). The bar charts represent mean parameter estimates with standard error bars in age group x conflict cluster.

There was a significant interaction between age group and conflict in the bilateral parietal cortex, right middle frontal gyrus (MFG) and right putamen. This was driven by increased activation in these regions in adults compared with adolescents in no-conflict compared to conflict trials (see Figure 4(a & b), Table 4).

**Table 4.** Conflict > no-conflict (adolescents > adults) (voxel-level uncorrected p < 0.001, cluster level corrected at p_FWE_< 0.05).

				Peak Voxel (in mm)		
Cluster	Brain Region	Size (N voxels)	z	**x**	**y**	**z**
Left Parietal	**Inferior Parietal Lobule (IPL)**	**68**	**5.48**	**−45**	**−46**	**56**
Right Parietal	**Postcentral Gyrus (PCG)**	**56**	**4.08**	**39**	**−37**	**50**
Right MFG cluster	**Middle Frontal Gyrus (MFG)**	**60**	**4.02**	**39**	**2**	**59**
	Superior Frontal Gyrus (SFG)		3.24	27	−1	68
Right Putamen	**Putamen**	**162**	**3.98**	**33**	**−10**	**−1**
	Superior Temporal Lobe		3.87	60	−28	11
	Thalamus		3.85	18	−13	2

##### Interaction between age group and social influence group

No significant cluster was found in the interaction between age groups and social influence group.

##### Interaction between age group, conflict and social influence group

No significant cluster was found in the interaction between age groups, conflict and social influence group.

##### Region of interest analysis: Nucleus accumbens

We found a significant interaction for social influence group x conflict in the NAcc (F (1, 34) = 35.412, p < 0.01). A *post hoc* test with Bonferroni alpha correction showed that there was a greater NAcc response to *adult* ratings in *no-conflict* trials (t(1) = 2.12, p < 0.05) and a greater response to *teenage* ratings in *conflict* trials (t(1) = 2.69, p < 0.05). No differences between the age groups were observed – there was no significant three-way interaction between social influence group, conflict and age group (F (1, 34) = 0.002, n.s.).

## Discussion

In the current fMRI study, we investigated the neural mechanisms underlying the relationship between social influence and risk perception in adolescents and adults. Participants were asked to rate the riskiness of everyday scenarios and were then informed about the risk ratings for the same scenarios from a social influence group, either *teenagers* or *adults*. When presented with a risk rating that conflicted with their initial risk rating of the same scenario, participants changed their risk rating significantly in the direction of the social influence group’s rating. Furthermore, when presented with conflicting information, this social influence effect was significantly greater in adolescents than in adults, in line with our previous findings (Knoll et al., 2017, 2015). Our fMRI results revealed that, when comparing *conflict* and *no-conflict* condition, a discrepancy between the participants’ initial rating and the ratings of the social influence group was associated with increased activation of the pMFC, dorsal ACC, IFG, and AI, in both adolescents and adults. Compared with adolescents, adults’ response to *no-conflict* trials was associated with increased activity in the middle frontal gyrus, the inferior parietal cortex and the right putamen. Additionally, the NAcc, which plays a key role in motivation and reward processing (Liu, Hairston, Schrier, & Fan, 2011; Sescousse, Caldú, Segura, & Dreher, 2013), was sensitive to the different social influence groups in conflict situations in both participant groups. Our behavioral findings provide evidence that adolescents are particularly susceptible to social influence. However, adolescents’ sensitivity to social conflict was not reflected in differences in neural activations between adolescents and adults.

### The social influence effect

In line with our first hypothesis (H1), the behavioral data showed that adolescents changed their second rating significantly more toward the rating of social influence groups than adults, irrespective of whether the social influence group were *teenagers* or *adults*. The decrease in social influence from adulthood to adolescence is in line with previous behavioral studies (Knoll et al., 2017, 2015). Our first hypothesis was based on our previous studies in which we found adolescents to be more influenced by the *teenage* social influence group. In contrast to these, the current study found that both age groups, adolescents and adults, were more influenced by *adults* than by *teenagers*. The reason for this conflicting finding is unclear, but it might be related to the fact that the current sample was significantly smaller (18 compared to over 60 young adolescents) than in our previous samples, and that the task took place in an MRI scanner. Another factor could be the different gender of participants in our studies: our previous studies included both male and female participants, whereas only female participants took part in the current neuroimaging study.

In the current study, participants changed their rating toward the ratings of the social influence group when their initial rating was in conflict with other people’s ratings, perhaps indicating a reevaluation of the situation in light of other people’s views. Examples of factors that could influence participants to reconsider their initial ratings could be trustworthiness, likability, or experience of the social influence group. In a previous study (Knoll et al., 2017), we found that children and young adolescents were more influenced by *teenage* feedback when the teenage social influence group rated a situation as riskier than they did. Children and young adolescents did not show a preference for the *teenage* feedback when the provided rating was less risky than their own rating. One reason for this observation might be that children and young adolescents place different values on the opinions of teenagers and adults than do older age groups, and it suggests that stereotypes about social influence groups, such as risk-prone teenagers, might interact with social influence.

### Neuroimaging results – social conflict

In line with our second hypothesis (H2), a conflict in rating with the social influence group was associated with activation in the pMFC, in addition to the dorsal ACC and IFG (shown in Figure 3(a)). Activation in the pMFC in response to social influence has been previously reported in studies investigating social conformity. For example, Klucharev et al. (2009) tested how participants’ ratings were influenced by others in a face attractiveness rating task. They found activation within the pMFC was elevated when participants were confronted with other people’s ratings that differed from their own rating. Results of a meta-analysis by Ridderinkhof and colleagues (2004) reported increased activation in the pMFC in studies investigating reaction to unfavorable outcomes, response conflict, and decision uncertainty. Additionally, pMFC activation is associated with behavioral change after cognitive dissonance (Izuma et al., 2010; van Veen, Krug, Schooler, & Carter, 2009). Izuma and colleagues (2010) asked participants to rate their preference on food items and, in a subsequent task, to choose between pairs of food; either between food items which they had previously given equal “liking” ratings, or between one “liked” and one “disliked” item. Participants were then asked to rate the food items again. The authors suggested that cognitive dissonance was increased when participants were given information that they had rejected a liked food during the choice phase when making their second rating. During these cognitive dissonance events, there was increased activation in a cluster comprising the dorsal ACC and pMFC. In the current study, both these areas were activated when participants saw that their initial rating was in conflict with the social influence group rating. The subsequent change in rating following this conflict could thus be motivated by the attempt to reduce cognitive dissonance. Furthermore, conflicting situation was associated with activation in the AI. Within social contexts, the AI-dorsal ACC network has been associated with experiences of social exclusion and rejection (Eisenberger & Lieberman, 2004; Eisenberger, Lieberman, & Williams, 2003; Layden et al., 2017). Thus, the activation patterns in during social conflict in the current study are consistent with previous imaging studies on social exclusion and cognitive dissonance.

### Neuroimaging results – social agreement

In our third hypothesis (H3), we predicted that agreeing with the social influence group would be reflected in activation in reward processing regions, such as NAcc, which plays a key role in motivation, reward and reinforcement processes (Liu et al., 2011; Sescousse et al., 2013), as has been found in previous studies (Klucharev et al., 2009). Klucharev and colleague (2009) observed more activity in no-conflict than conflict trails in ratings on facial attractiveness in the NAcc. However, we found no evidence for activation of reward-related brain regions in *no-conflict* situations in a whole brain analysis or in a region of interest analysis focussed on the NAcc. It is possible that agreeing with others on the perceived degree of risk is not salient enough to be perceived as rewarding, and that conforming *action* rather than conforming *perception* is required to drive sizable reward-related activity. However, we observed a significant interaction in the NAcc for social influence group and conflict: *no-conflict* trials evoked a greater response when the *adult* ratings were shown and *conflict* trials evoked a greater response when *teenage* ratings were presented. In addition, previous studies have suggested that NAcc is hypersensitive to reward in adolescence, and that activity in the NAcc peaks during adolescence (Braams et al., 2015). However, in the current study, we did not find any age-related differences in activation in the NAcc (H3). The lack of observed age differences in the NAcc suggests that reward hypersensitivity in adolescence might be context-dependent. In fact, this echoes some previous behavioral studies, where it has been found that risk-taking in adolescence highly dependent on the social context (Gardner & Steinberg, 2005), whereas non-affective gambling tasks do not always show age differences in risky behavior (e.g. Wolf, Wright, Kilford, Dolan, & Blakemore, 2013). The current results suggest that social agreement is not sufficient to evoke activity in the NAcc in either age group.

Our study revealed activations in parietal regions and the posterior insular as well as the PCC across all participants during social agreement. These areas are involved in multiple social cognitive processes, for example the PCC is activated by the evaluation of others’ intentions (Behrens, Hunt, & Rushworth, 2009; Smith, Clithero, Boltuck, & Huettel, 2014) and in various self-related aspects of cognitive processing (Johnson et al., 2002; Schulte-Rüther, Markowitsch, Fink, & Piefke, 2007), processes which might come into play during the reevaluation of risk in the current study.

### Neuroimaging results – social influence groups

In our fourth hypothesis (H4), we predicted differences in the processing of feedback provided by the social influence groups on the reevaluation of ratings in *conflict* and *no-conflict* situations; for example in the dmPFC, which is sensitive to behavioral adjustments from disliked groups toward liked groups (Izuma & Adolphs, 2013). Our behavioral findings suggested that participants distinguished between the provided information of the *teenage* and *adult* social influence group. As discussed above, in *conflict* situations, both adolescents and adults changed their ratings more in the direction of the ratings of *adults* than *teenagers*. However, these behavioral findings were not reflected in different activation patterns. There was increased activation in the right amygdala in response to the social influence group *teenagers* compared with *adults* in both age groups, irrespective of whether the social influence group’s ratings were in conflict or agreement with participants’ initial ratings. Previous studies have demonstrated the importance of the amygdala in social behavior (Phelps & LeDoux, 2005) and the evaluation of emotional stimuli (Phelps & Anderson, 1997). It is unclear why activation in the amygdala was increased when participants were provided with the ratings of *teenagers*. However, previous studies have observed amygdala activation not only to high arousal stimuli, but also to stimuli that are interesting and unusual (Hamann, Ely, Hoffman, & Kilts, 2002).

### Neuroimaging results – age-related differences

Our behavioral results showed that, compared with adults, adolescents were more sensitive to social conflict and adapted their ratings accordingly. However, this age-related behavioral difference was not paralleled by differences in brain activation in adolescents compared to adults. Age-related differences in brain activation to conflict versus no-conflict conditions were observed in the middle frontal gyrus (MFG), the inferior parietal cortex and the right putamen, but this interaction between age group and conflict was driven by adult participants when their initial rating was in agreement with the social influence groups. Activation in these regions, particularly the bilateral activation in the parietal cortex, has been found in studies investigating social cognition (Bzdok et al., 2016). We observed similar activation patterns in the main effect of social agreement (vs conflict; see previous paragraph), suggesting that this activation was mostly driven by the adult participants and that neural mechanisms associated with agreement are still maturating during adolescence.

### Self-reported resistance to peer influence (RPI)

In addition, we explored individual differences in self-reported resistance to peer influence. Contrary to our prediction, there was no difference between adolescents’ and adults’ total score on the RPI questionnaire. The mean score in our adolescent sample was comparable to the original study (Steinberg & Monahan, 2007), but the adult sample scored lower than expected. The original study included more than 3500 participants age 10 to 30, considerably larger than the sample size of the current study. It is possible that the RPI measure is not sufficiently sensitive to reliably detect age differences in peer influence on risk taking in relatively small samples.

### Sex and gender differences in social influence

A future fMRI study should explore the effect of sex or gender on social influence. The current study included only female participants to avoid developmental (e.g. pubertal) sex differences. Previous research has shown inconsistent results in terms of sex or gender differences in social influence. Several studies have not found any effect of gender on social influence (Berns et al., 2010; Steinberg & Monahan, 2007). One study found that girls are less susceptible to social influence (Steinberg & Silverberg, 1986), but other studies have indicated that girls are more susceptible to implicit social influence such as pressure to conform and follow norms than boys are (Iscoe, Williams, & Harvey, 1963). Boys appear to be more affected by explicit and overt attempts of pressure from their peers (Berndt, 1979). In our study, the provided rating of the social influence group can be considered as implicit social influence, which could have a stronger influence effect on female participants compared to male participants. This could be explored in future studies.

## Conclusion

This neuroimaging study sought to replicate previously established behavioral effects of social influence and to explore their underlying neural mechanisms in adolescents and adults. We were especially interested in the processing of social conflict and social agreement, and how that might change with age. At the behavioral level, adolescents showed a greater sensitivity to conflict between their own rating and the rating of others compared with adults. At the neural level, when seeing risk ratings (from either social influence group) that conflicted with their own, both age groups showed increased activation in the pMFC and dACC, providing evidence for the sensitivity of these regions to social conflict. Finally, adults showed a greater activation in the MFG and the parietal cortex during social agreement compared with adolescents, highlighting an age-related difference in the neural mechanisms that process agreement, rather than conflict, under social influence.

## Data Availability

The data that support the findings of this study are available from the corresponding author upon request.
